# Safety, tolerability and pharmacokinetics of WXFL10203614 in healthy Chinese subjects: A randomized, double-blind, placebo-controlled phase Ⅰ study

**DOI:** 10.3389/fphar.2022.1057949

**Published:** 2022-11-04

**Authors:** Kai Huang, Ying Ding, Linling Que, Nannan Chu, Yunfei Shi, Zhenzhong Qian, Wei Qin, Yuanxin Chen, Xianghong Gu, Jiakun Wang, Zhiwei Zhang, Jianguo Xu, Qing He

**Affiliations:** ^1^ Drug Clinical Trial Institution, Affiliated Wuxi People’s Hospital of Nanjing Medical University, Wuxi, China; ^2^ Wuxi Fuxin Pharmaceutical Research and Development Co, Ltd, Wuxi, China

**Keywords:** WXFL10203614, pharmacokinetics, JAK1 inhibitor, rheumatoid arthritis, first-in-human

## Abstract

**Objective:** This study was conducted to investigate the safety, tolerability and pharmacokinetics (PK) of WXFL10203614 after single and multiple oral doses in healthy Chinese subjects.

**Methods:** A single-center, randomized, double-blind, placebo-controlled phase Ⅰ study was performed on healthy Chinese subjects. In the single-dose study, Subjects were randomized into 7 dose levels of WXFL10203614 (1 mg group, *n* = 2; 2, 5, 10, 17, 25 and 33 mg groups with placebo, 8 subjects per group, 2 of them given placebo). In the multiple-dose study, subjects received 5 or 10 mg WXFL10203614 once daily (QD), 5 mg twice daily (BID) or placebo for 7 consecutive days. Safety, tolerability and PK of WXFL10203614 were all assessed.

**Results:** A total of 592 subjects were screened, 50 subjects were enrolled in the single-dose study and 30 in the multiple-dose study. All adverse events (AEs) were mild or moderate and resolved spontaneously. No Serious Adverse Events (SAEs) or deaths were reported during the study. WXFL10203614 was absorbed rapidly after dosing with T_max_ of 0.48–0.98 h, C_max_, AUC_0-t_ and AUC_0-∞_ were all increased in a dose-related manner over the range of 1–33 mg. Renal excretion was the major route of elimination of WXFL10203614. Steady-state PK parameters (C_max,ss_, AUC_0-t,ss_ and AUC_0-∞,ss_) were elevated after once-daily administration of 5–10 mg WXFL10203614 and non- and weak drug accumulations were observed, whereas moderate drug accumulation occurred in the 5 mg BID group.

**Conclusion:** WXFL10203614 exhibited good safety, tolerability and favorable PK profiles in healthy Chinese subjects, supporting further clinical development in patients with rheumatoid arthritis.

**Clinical Trials Registration Number:** http://www.chinadrugtrials.org.cn/index.html, #CTR20190069 and CTR20200143.

## Introduction

Rheumatoid arthritis (RA) is a chronic systemic autoimmune disease. It is characterized by joint synovitis, bone erosion, progressive cartilage destruction and disability, which seriously influence life quality and even increase mortality (Mohamed et al., 2016; Sparks, 2019). So far, however, the exact etiology of RA has still been unclear (Li et al., 2020).

Although significant progress has been made in non-steroidal anti-inflammatory drugs (NSAIDs), conventional synthetic disease-modifying anti-rheumatic drugs (csDMARDs) and biological therapeutics for the treatment of RA, some shortcomings were still inevitable during the treatments, such as inadequate or no response to traditional therapies, serious complications of infection, inconvenient injection and high cost of biological therapy, which limited their long-term use (Lin et al., 2020; Xie et al., 2020). A more effective and better tolerated oral treatment may be preferable for RA patients. A great deal of attention has been paid to the Janus kinase (JAK) family when JAK-mediated signal pathways were confirmed to play a pivotal role in the pathologic processes of RA (Norman, 2014; Alqarni and Zeidler, 2020; You et al., 2020). Therefore, inhibition of JAK isoforms was considered a potential therapeutic approach for RA.

JAK family comprises JAK1, JAK2, JAK3 and tyrosine kinase 2 (TYK2), mediating the signal pathways of numerous cytokines and growth factors which participate in the regulation of immune function, inflammation and hematopoiesis (Vazquez et al., 2018). However, among 4 JAK isoforms, JAK1 mainly regulated various cytokine signal pathways related to the pathophysiology of multiple inflammatory diseases (Kotenko and Pestka, 2000; Peeva et al., 2018), JAK2 was involved in the essential regulatory functions of granulocyte-macrophage colony-stimulating factor (GM-CSF) and erythropoietin (Broxmeyer, 2013), JAK3 was more selectively expressed in hematopoietic cells and Tyk2 was a target for treating psoriasis and inflammatory bowel disease rather than RA (Norman, 2014). Immunoregulation and anti-inflammation were the keys for JAK inhibitors in RA treatment *via* inhibiting the JAK-mediated signal pathways. A series of small molecule inhibitors of JAK isoforms have been approved for treating RA since 2009 (Choy, 2019), including tofacitinib (the first JAK1/JAK3 inhibitor), baricitinib (JAK1/JAK2 inhibitor), upadacitinib and filgotinib (JAK1 inhibitor) and peficitinib (pan-JAK inhibitor) (Norman, 2014; Kaneko, 2020). Therefore, developing selective JAK1 inhibitors not only minimizes the potential side effects but also maximizes the therapeutic efficacy for RA patients.

WXFL10203614, a potential selective JAK1 inhibitor, was under development as an oral DMARD for RA treatment. The preclinical studies confirmed that WXFL10203614 was effective for RA with acceptable safety and tolerability profiles and superior to tofacitinib (data not shown). Based on these promising preclinical data, the clinical trials of WXFL10203614 were eventually approved by National Medical Products Administration (NMPA) in 2018 (Approval Number: 2018L03083 and 2018L03084).

This single- and multiple-dose study aimed to evaluate the safety, tolerability and pharmacokinetics (PK) of WXFL10203614 in healthy Chinese subjects. Results supported the dose selection and design of phaseⅡclinical trials of WXFL10203614 in RA patients.

## Methods

### Study population

Healthy Chinese subjects aged 18–45 years, with a body mass index of 19.0–26.0 kg/m^2^ and body weight ≥50 kg (male) or ≥45 kg (female), were enrolled in this study. Subjects were included if they were evaluated to be healthy by vital signs, physical examination, medical history, laboratory tests, virological examinations, electrocardiograph (ECG), chest X-ray, abdominal ultrasound, urine nicotine test, alcohol breath analysis and urine drug screening test within 2 weeks before dosing. Subjects were excluded if they had any of the following: allergic to any medication, a history of corrected Q-T (QTc) interval prolongation, QTc interval ≥450 milliseconds, PR interval ≥210 milliseconds, QRS ≥120 milliseconds, positive for T-SPOT^®^ TB test, took any prescription or nonprescription medicine (including vitamins, herbal products or dietary supplements) within 1 month before the screening, or participated in any clinical trial within the past 3 months. Besides, pregnant or lactating women were also excluded.

### Compliance with ethical standards

This study was conducted according to Good Clinical Practice guidelines and the ethical principle of the Declaration of Helsinki. The study protocol and informed consent form were approved by the independent Ethics Committee of Affiliated Wuxi People’s Hospital of Nanjing Medical University (Approval Number: 2018LLPJ-I-45 for the single-dose study and 2020LLPJ-I-08 for the multiple-dose study). The written informed consent was signed by each subject freely and voluntarily before the screening.

### Study design

A single-center, randomized, double-blind, placebo-controlled, single- and multiple-dose phase Ⅰ study of WXFL10203614 was conducted in the phase Ⅰ center of Wuxi people’s hospital from 7 March 2019 to 13 December 2019 (the single-dose study) and from 31 March 2020 to 10 July 2020 (the multiple-dose study) (Chinese Clinical Trial Registry, Registration Number: CTR20190069 and CTR20200143, http://www.chinadrugtrials.org.cn/index.html). The WXFL10203614 tablet (1 mg, Lot Number: XS180902B; 5 mg, Lot Number: XS180902C) and the placebo tablet (Lot Number: XS180901A) were manufactured and supplied by Wuxi Fuxin Pharmaceutical Research and Development Co., Ltd.

In the single-dose study, 50 subjects were randomly allocated to receive a single ascending dose of 1, 2, 5, 10, 17, 25 and 33 mg WXFL10203614. In the initial dose cohort, 2 subjects received 1 mg WXFL10203614 only for the safety evaluation. After that, 8 subjects per cohort randomly received WXFL10203614 (2, 5, 10, 17, 25 or 33 mg) or placebo at a 3:1 ratio in a dose-escalation manner. All subjects fasted overnight for at least 10 h before dosing and received a single oral dose of WXFL10203614 with 240 ml water. Moreover, none of the subjects was permitted to drink water within 1 h before and after dosing or eat anything within 4 h after dosing. Subjects remained in the center for 4 days and completed follow-up on day 7 after discharge. Safety, tolerability and PK evaluations were performed before escalation to the next dose level.

In the multiple-dose study, three doses of WXFL10203614 (5 or 10 mg once daily [QD] or 5 mg twice daily [BID]) were selected on the basis of the single-dose study. 30 subjects were enrolled, 10 subjects per cohort were randomly assigned to receive WXFL10203614 or placebo at a 4:1 ratio for 7 consecutive days (QD: once daily on day 1–7; BID: once daily on day 1 and 7, and twice daily on day 2–6). Subjects fasted overnight before the first and last dosing and orally received WXFL10203614 with 240 ml water at each dosing time. Subjects remained in the center until day 10 and completed follow-up on day 7 after discharge. Safety, tolerability and PK were also evaluated at the end of the study. Dose escalation was only allowed when the previous dose was safe and well-tolerated in subjects.

### Safety and tolerability evaluations

Safety and tolerability were assessed on the basis of Adverse Events (AEs), the clinically significant changes in physical examination, vital signs, laboratory tests and ECG. The severity of AEs was referenced in terms of the National Cancer Institute- Common Terminology Criteria for Adverse Events (NCI-CTCAE) (version 5.03). Furthermore, the relationship of AEs to WXFL10203614 was evaluated by the investigator.

### Pharmacokinetic analysis

In the single-dose study, blood samples (4 ml) were collected in Ethylene Diamine Tetraacetie Acid (EDTA)-2K vacuum tubes at 0 (pre-dose), 0.25, 0.5, 1, 1.5, 2, 3, 4, 6, 8, 12, 24, 36, 48 and 72 h. Urine samples were collected at -2-0 h (pre-dose), 0–4, 4–8, 8–12, 12–24, 24–36, 36–48 and 48–72 h. In the multiple-dose study, blood samples (4.0 ml) were collected on day 1 at the same points as in the single-dose study until 24 h (before the second dosing) and on day 5 and 6 before dosing. Moreover, the sampling time points were 0 (pre-dose), 0.25, 0.5, 0.75, 1, 1.5, 2, 4, 8, 12, 24, 36 and 48 h after the last dosing on day 7. Blood samples were gently mixed 6–8 times after collecting and centrifuged at 1500 g for 10 min at 4°C. All plasma and urine samples were stored at −80°C until analyzed.

Samples were prepared by simple protein precipitation with acetonitrile containing ^13^CD_3_-Target as internal standard (IS). Briefly, 50 μL of IS working solution (50 ng/ml for plasma or 200 ng/ml for urine) and 300 μl of acetonitrile were added to 50 μl of the sample. The mixture was vortexed for 15 min, followed by centrifugation at 2,510 *g* for 15 min. The supernates were assayed for the WXFL10203614 concentration in plasma. For the urine sample, the supernate should be diluted 10-fold before analysis.

The concentrations of WXFL10203614 in plasma and urine were analyzed using a validated liquid chromatography-tandem mass spectrometry (LS-MS/MS) method. WXFL10203614 and IS were chromatographed by injecting 1 μl sample into a BEH C18 column (1.7 μm, 2.1 mm × 50 mm, Waters, USA) with a column temperature at 40°C. The mobile phase consisted of solvent A (0.05% formic acid in a 2 mM aqueous ammonium acetate solution) and solvent B (acetonitrile). The gradient elution was programmed as follows: 90% solvent A for 0.3 min, and then gradually decreased to 5% for 1.4 min, followed by re-equilibration at 90% for 0.8 min. The total analytical run time was 2.5 min and the flow rate was 0.55 ml/min. The mass transitions of WXFL10203614 and IS monitored in multiple reaction monitoring (MRM) were m/z 294.1→146.0 and 298.2→146.1. The retention times of WXFL10203614 and IS were 1.06 and 1.05 min, and no apparent interference affected the detection. The linearity ranges of WXFL10203614 were 0.4–500 ng/ml and 5–8,000 ng/ml, the lower limit of quantitation (LLOQ) was 0.4 and 5 ng/ml, and the recoveries were 90.9–94.2% and 92.0–94.0% in plasma and urine, respectively. The intra-and inter-day precision did not exceed 6.1% with an accuracy of −4.2-2.9% in plasma (Low QC: 1.2 ng/ml, Middle QC: 50 ng/ml and High QC: 400 ng/ml). The plasma samples were stable at room temperature for 18 h, and −20°C and −80°C for 105 days. The intra-and inter-day precision did not exceed 10.7% and the accuracy was within ±7.2% in urine (Low QC: 15 ng/ml, Middle QC: 500 ng/ml and High QC: 6,400 ng/ml). The urine samples were stable at room temperature for 24 h, and −20°C and −80°C for 69 days. The concentrations of WXFL10203614 in plasma or urine that exceed the upper limit of quantification (ULOQ) should be diluted, and 10-fold dilutions did not affect the accuracy and precision.

### Statistical analysis

PK analysis was performed by a non-compartmental method using Phoenix WinNonlin software (version 8.2) to calculate PK parameters. In the single-dose study, the primary PK parameters included maximum concentration in plasma (C_max_), time to maximum concentration (T_max_), area under the plasma concentration-time curve from time 0 to the last measurable concentration (AUC_0-t_), area under the plasma concentration-time curve from time 0 to infinity (AUC_0-∞_), terminal elimination half-life (t_1/2_), mean residence time of 0 to the last measurable concentration (MRT_0-t_), apparent clearance rate (CL/F), apparent volume of distribution (Vd) and the cumulative urinary excretion in 72 h (Ae_0-72_). In the multiple-dose study, the primary PK parameters were C_max_ at steady state (C_max,ss_), Tmax at steady state (T_max,ss_), AUC_0-t_ at steady state (AUC_0-t,ss_), AUC_0-∞_ at steady state (AUC_0-∞,ss_), CL/F at steady state (CL_ss_/F) and the accumulation index (Ra). Ra (AUC) was calculated as the ratio of AUC_0-t,ss_ to AUC_0-t_ and Ra (C_max_) was the ratio of C_max,ss_ to C_max_.

The Full Analysis Set (FAS) and Safety Set (SS) included all randomized subjects receiving at least one dose of WXFL10203614 or placebo. The pharmacokinetic concentration set (PKCS) included all subjects with at least one drug concentration result. The pharmacokinetic parameter set (PKPS) included all subjects with at least one viable PK parameter.

Dose-PK parameter (C_max_, AUC_0-t_ and AUC_0-∞_) proportionality was assessed with linear regression by using natural logarithm value based on the power model. The corresponding 90% confidence intervals (CIs) for the slopes of the log-transformed C_max_, AUC_0-t_ and AUC_0-∞_ were compared with the modified acceptance range (Smith et al., 2000). PK parameters and demographic characteristics were summarized using descriptive statistics. All statistical analyses were performed with SAS software (version 9.4). Variance analysis and *t*-test for normally distributed data and Wilcoxon rank test for non-normally distributed data were used for data analysis. *p* < 0.05 was considered statistically significant.

## Results

### Demographic data of subjects

**FIGURE 1:**
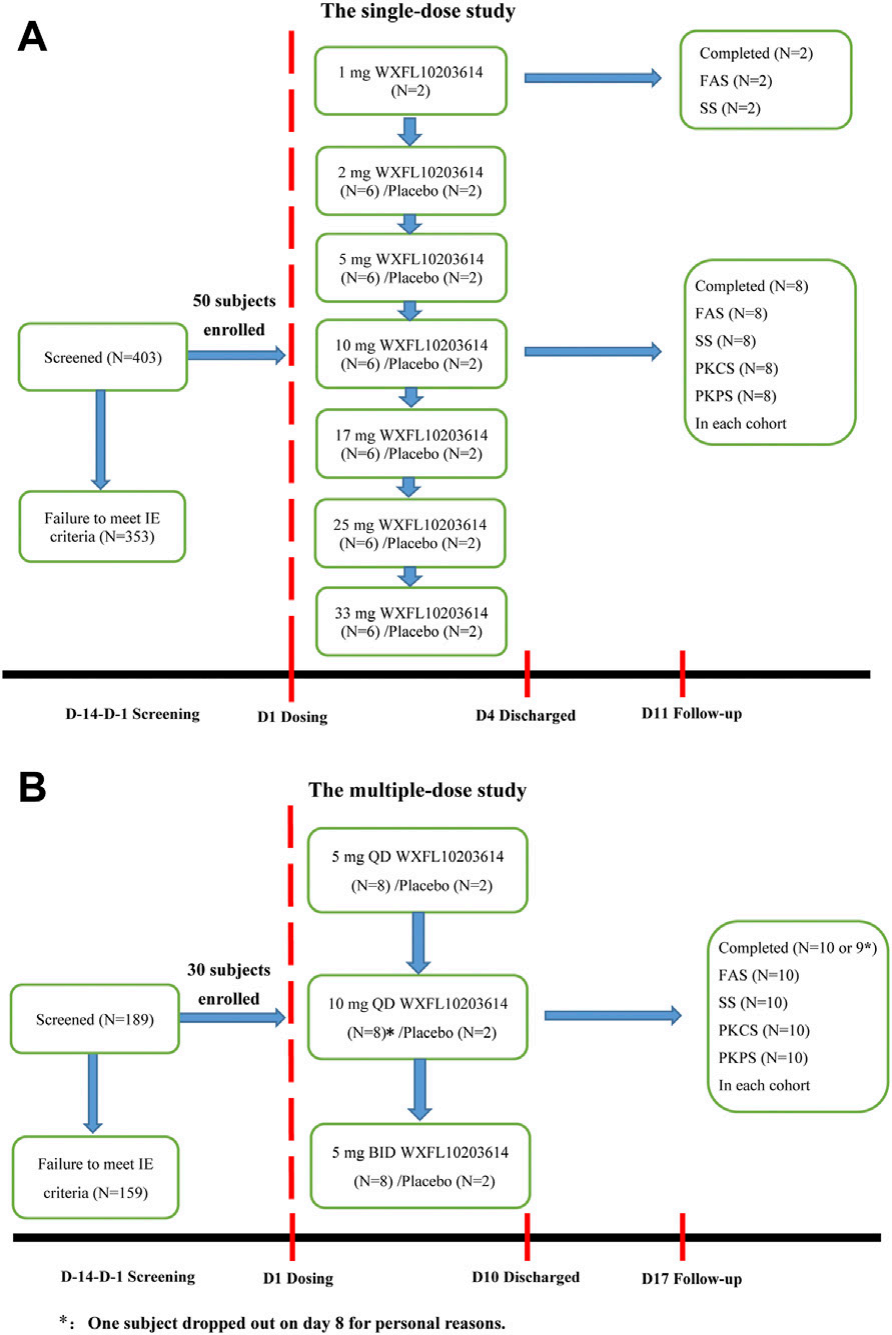
Subject disposition. **(A)** The single-dose study, **(B)** The multiple-dose study. FAS: The Full Analysis Set, SS: Safety set, PKCS: Pharmacokinetic concentration set, PKPS: Pharmacokinetic parameter set, IE: inclusion/exclusion.

In the single-dose study, 403 subjects were screened, 50 healthy subjects randomized and exposed to oral administration. Among them, 32 were male and 18 were female, all subjects completed the study. Two subjects who received 1 mg WXFL10203614 were only evaluated for FAS and SS, and others were included in FAS, SS, PKCS and PKPS analyses. In the multiple-dose study, 189 subjects were screened, 30 subjects were enrolled and randomized, 18 were male and 12 were female, 29 subjects completed the study and 1 subject who received 10 mg QD WXFL10203614 dropped out on day 8 for personal reasons. All subjects were included in FAS, SS, PKCS and PKPS analyses (Figure 1). Age, weight and BMI were similar between WXFL10203614 groups and placebo in the single- and multiple-dose study. Baseline demographic characteristics are shown in Table 1.

**TABLE 1:** Demographics and baseline characteristics of subjects.

	Single-dose study								Multiple-dose study			
	1 mg (N = 2)	2 mg (N = 6)	5 mg (N = 6)	10 mg (N = 6)	17 mg (N = 6)	25 mg (N = 6)	33 mg (N = 6)	Placebo (N = 12)	5 mg QD (N = 8)	10 mg QD (N = 8)	5 mg BID (N = 8)	Placebo (N = 6)
Sex, N (%)												
Male	1 (50.0)	4 (66.7)	2 (33.3)	4 (66.7)	4 (66.7)	5 (83.3)	4 (66.7)	8 (66.7)	3 (37.5)	5 (62.5)	6 (75.0)	4 (66.7)
Female	1 (33.3)	2 (33.3)	4 (66.7)	2 (33.3)	2 (33.3)	1 (16.7)	2 (33.3)	4 (33.3)	5 (62.5)	3 (37.5)	2 (25.0)	2 (33.3)
Ethnicity (Han/other)	2/0	5/1	6/0	6/0	6/0	6/0	6/0	12/0	8/0	7/1	8/0	6/0
Age (years)	22.5 ± 0.7	27.8 ± 4.2	28.0 ± 5.1	26.8 ± 6.9	32.0 ± 4.4	27.2 ± 6.6	28.7 ± 5.2	27.6 ± 5.9	24.3 ± 4.4	26.3 ± 5.4	32.8 ± 8.0	28.7 ± 7.1
Height (cm)	159.5 ± 7.1	162.5 ± 5.0	162.6 ± 7.9	170.7 ± 8.1	165.3 ± 8.4	168.6 ± 8.7	164.2 ± 8.6	164.7 ± 7.3	163.9 ± 10.9	168.8 ± 6.9	168.7 ± 7.1	166.2 ± 9.0
Weight (kg)	55.0 ± 2.3	59.9 ± 6.7	60.8 ± 6.7	65.5 ± 8.1	64.3 ± 7.9	64.2 ± 9.0	55.9 ± 5.9	59.2 ± 6.9	59.3 ± 8.5	62.6 ± 8.3	61.4 ± 7.7	60.6 ± 8.4
BMI (kg/m^2^)	21.6 ± 1.1	22.7 ± 2.1	23.0 ± 1.3	22.5 ± 2.4	23.5 ± 0.8	22.5 ± 1.2	20.8 ± 1.6	21.8 ± 1.9	22.0 ± 1.0	21.9 ± 1.6	21.5 ± 1.8	21.8 ± 1.7

BMI: body mass index; Data are presented in mean ± SD, unless otherwise indicated.

### Pharmacokinetic and statistical analyses

**FIGURE 2:**
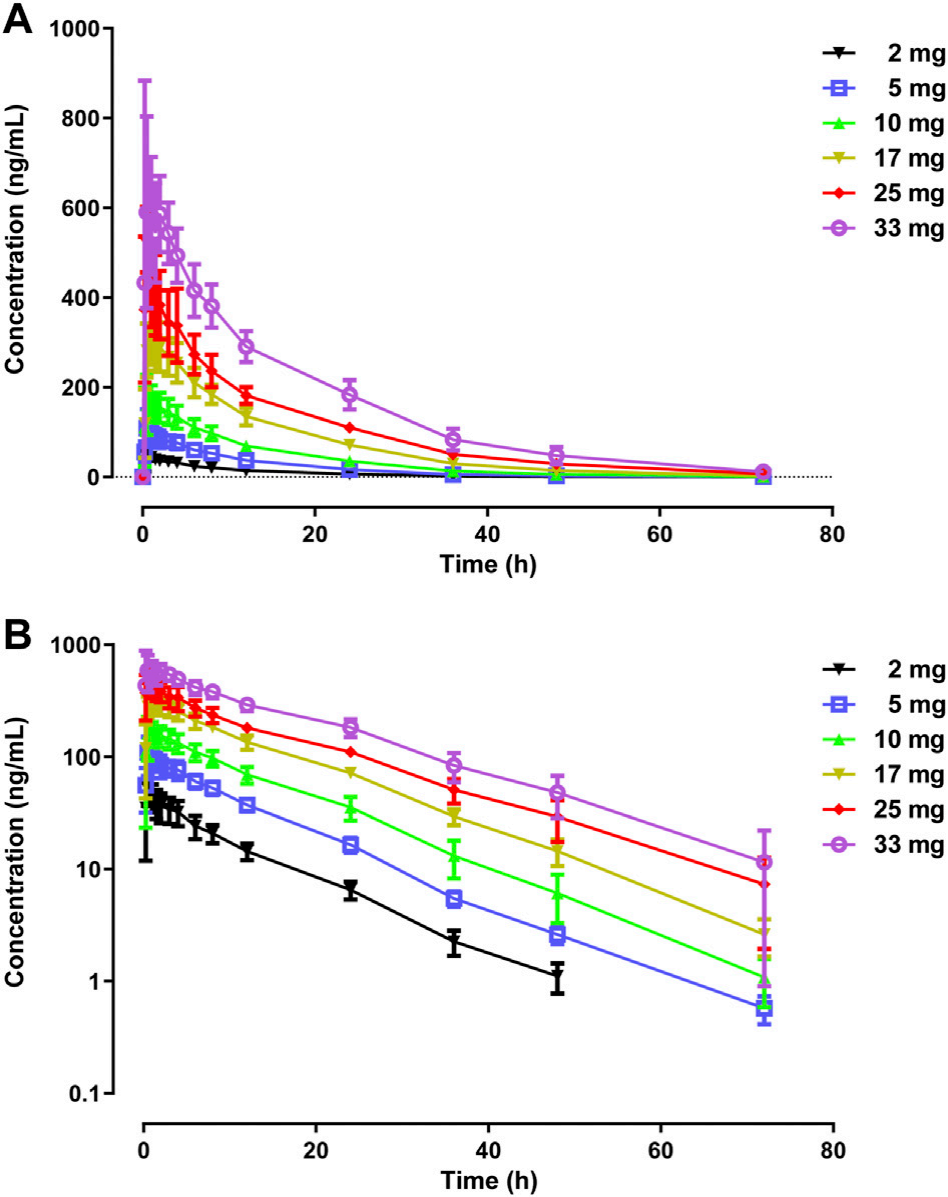
Mean plasma concentrations *versus* time profiles following a single dose of WXFL10203614 (2–33 mg) in healthy Chinese subjects on linear **(A)** and semilogarithmic scales **(B)**.

**FIGURE 3:**
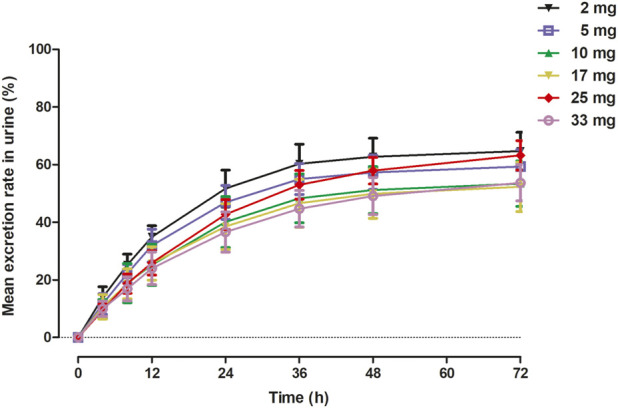
Mean excretion rate (%) *versus* time in urine in 72 h following a single dose of WXFL10203614.

The primary PK parameters and plasma concentration-time profiles of WXFL10203614 in the single-dose study are shown in Table 2 and Figure 2, respectively. WXFL10203614 was absorbed rapidly following single doses of 2–33 mg under fasted conditions and the median T_max_ was 0.48–0.98 h. CL/F ranged from 3.04 to 4.23 L/h, Vd was 52.9–63.1 L, t_1/2_ was 9.2–12.4 h, and MRT_0-t_ was 11.6–17.1 h which was slightly prolonged with the increasing doses of WXFL10203614. C_max_, AUC_0–t_ and AUC_0–∞_ were all increased across the dose range. However, the results of statistical analyses showed that the 90% CIs for the slopes of the log-transformed C_max,_ AUC_0-t_ and AUC_0–∞_ were not wholly contained within the acceptance range of 0.92–1.08 (C_max_:0.87–1.01, AUC_0-t_:1.04–1.12 and AUC_0-∞_:1.04–1.12), it was inconclusive that PK parameters were proportional to dose within the dose range (Smith et al., 2000). In addition, Ae_0-72_ ranged from 1.29 to 17.70 mg almost reaching the plateau of total cumulative urinary excretion and exceeding 50% of the administered dose in each group (Table 2 and Figure 3), suggesting that renal excretion was the main excretion route of WXFL10203614.

**TABLE 2:** The primary pharmacokinetic parameters of WXFL10203614 in the single-dose study.

Parameter	2 mg (N = 6)	5 mg (N = 6)	10 mg (N = 6)	17 mg (N = 6)	25 mg (N = 6)	33 mg (N = 6)
C_max_ (ng/ml)	52.9 ± 15.6	118.0 ± 19.7	192.0 ± 54.4	322.3 ± 61.0	577.0 ± 65.0	748.3 ± 276.9
T_max_ (h)^a^	0.48 (0.23–0.98)	0.48 (0.48–0.98)	0.98 (0.23–2.00)	0.73 (0.48–1.48)	0.48 (0.23–0.98)	0.98 (0.23–2.00)
AUC_0–t_ (h•ng/mL)	504 ± 94	1250 ± 183	2,400 ± 382	4,710 ± 455	6,940 ± 535	10800 ± 1580
AUC_0–∞_ (h•ng/mL)	518 ± 93	1270 ± 176	2,420 ± 385	4,760 ± 456	7,090 ± 596	11100 ± 1740
t_1/2_ (h)	9.2 ± 1.1	9.9 ± 1.7	9.5 ± 0.8	10.4 ± 1.1	12.4 ± 3.1	12.4 ± 3.8
MRT_0–t_ (h)	11.6 ± 1.2	12.6 ± 0.8	13.8 ± 2.1	15.0 ± 1.4	16.6 ± 2.7	17.1 ± 2.5
CL/F (L/h)	3.95 ± 0.65	4.02 ± 0.58	4.23 ± 0.65	3.60 ± 0.35	3.54 ± 0.29	3.04 ± 0.50
Vd (L)	52.9 ± 11.5	56.7 ± 6.4	57.2 ± 7.2	54.2 ± 7.3	63.1 ± 13.6	53.0 ± 11.4
Ae_0-72_ (mg)	1.29 ± 0.13	2.97 ± 0.30	5.34 ± 0.79	8.88 ± 1.45	15.80 ± 1.28	17.70 ± 2.09

^a^ Median (minimum, maximum).

**FIGURE 4:**
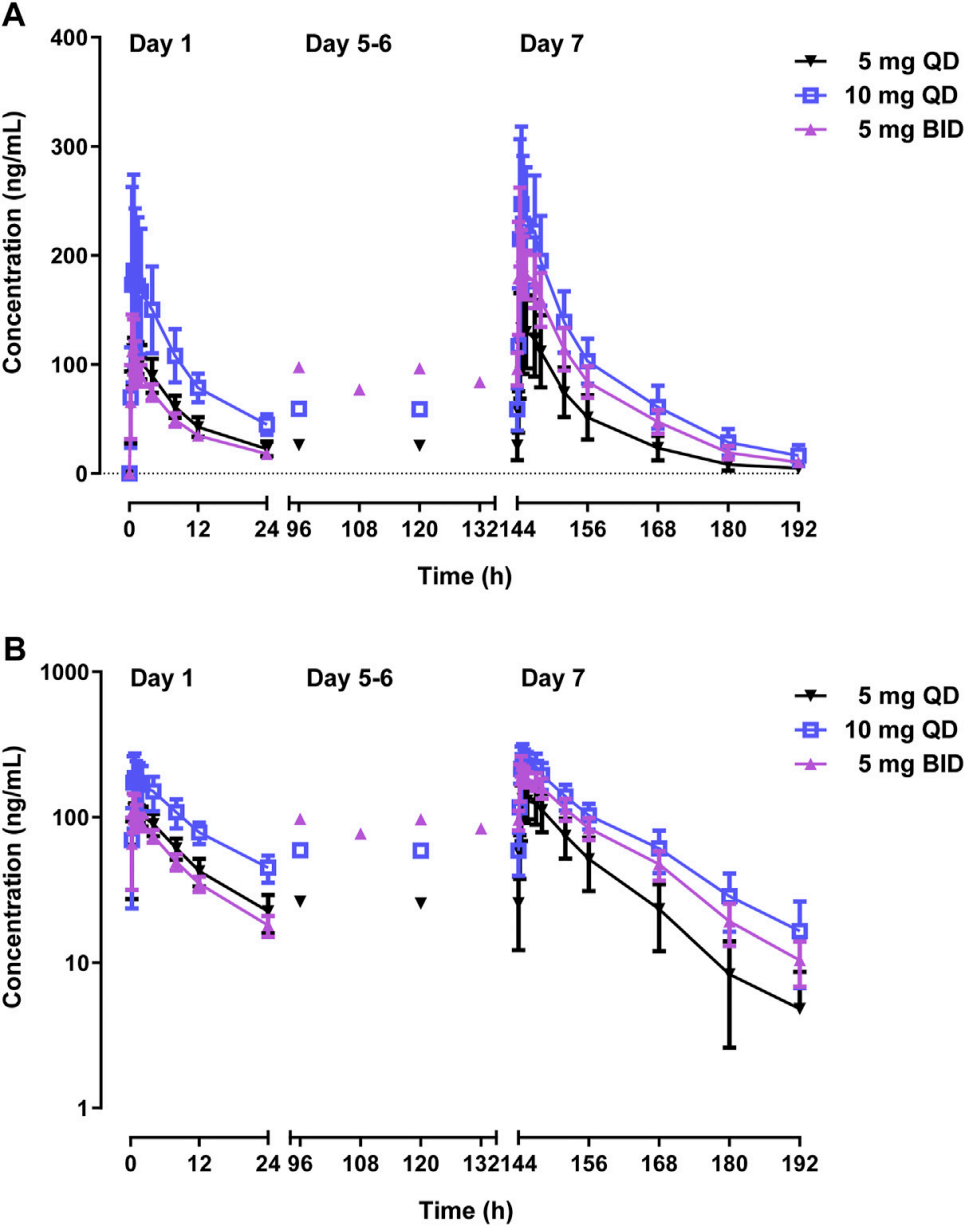
Mean plasma concentrations *versus* time profiles following multiple doses of WXFL10203614 (5 mg QD, 10 mg QD and 5 mg BID) in healthy Chinese subjects on linear **(A)** and semilogarithmic scales **(B)**.

Table 3 and Figure 4 shows PK parameters and plasma concentration-time profiles in the multiple-dose study. Similar to the single-dose study, WXFL10203614 rapidly reached peak concentrations (median T_max_ was 0.48–0.88 h) on day 1 following 5–10 mg QD WXFL10203614, the median T_max, ss_ and t_1/2_ was 0.73–1.23 h and 9.2–12.0 h on day 7, while CL_ss_/F remained constant (3.6 L/h) regardless of the dose. Steady-state trough concentrations were 26.2 ± 11.8, 25.5 ± 13.4 and 25.6 ± 13.4 ng/ml for 5 mg QD WXFL10203614, as well as 59.3 ± 15.5, 58.8 ± 17.9 and 58.9 ± 19.4 ng/ml for 10 mg QD WXFL10203614 in day 5, 6 and 7 mornings, indicating that the steady state of WXFL10203614 in plasma has been reached after multiple doses. AUC_0–t,ss_ and C_max,ss_ were increased with the dose ranging from 5 to 10 mg. Ra (AUC) and Ra (C_max_) were 1.17–1.34 and 1.14–1.37, respectively. In the 5 mg BID group, Steady-state trough concentrations of WXFL10203614 in 3 days were 97.5 ± 12.6, 96.5 ± 13.9 and 95.8 ± 14.9 ng/ml, which confirmed the steady-state trough concentration was stable. The median T_max, ss_ was 0.48 h and t_1/2_ was 11.1 h, Ra (AUC) and Ra (C_max_) were 2.17 ± 0.15 and 1.85 ± 0.21, indicating that the obvious accumulation might occur following multiple doses of 5 mg BID WXFL10203614.

**TABLE 3:** The primary pharmacokinetic parameters of WXFL10203614 on day 1 and 7 in the multiple-dose study.

	Parameter	5 mg QD (N = 8)	10 mg QD (N = 8)	5 mg BID (N = 8)
Day 1	C_max_ (ng/ml)	126.0 ± 16.8	216.0 ± 86.6	124.0 ± 24.6
	T_max_ (h)^a^	0.48 (0.48–1.00)	0.88 (0.48–3.98)	0.50 (0.48–1.48)
	AUC_0–12_ (h•ng/ml)	886 ± 132	1500 ± 397	753 ± 88
	AUC_0–24_ (h•ng/ml)	1260 ± 218	2,210 ± 489	1060 ± 125
Day7	C_max, ss_ (ng/ml)	143.0 ± 42.0	264.0 ± 72.8	226.0 ± 36.1
	T_max, ss_ (h)^a^	1.23 (0.50–1.98)	0.73 (0.48–1.48)	0.48 (0.48–0.50)
	AUC_0–12, ss_ (h•ng/ml)	1080 ± 319	1960 ± 400	1640 ± 255
	AUC_0–24, ss_ (h•ng/ml)	1500 ± 500	2,920 ± 602	2,400 ± 402
	AUC_0–t, ss_ (h•ng/ml)	1750 ± 651	3,620 ± 936	2,950 ± 549
	AUC_0–∞, ss_ (h•ng/ml)	1810 ± 711	3,940 ± 1080	3,110 ± 618
	t_1/2_ (h)	9.2 ± 1.6	12.0 ± 2.6	11.1 ± 1.1
	CLss/F (L/h)	3.6 ± 1.0	3.6 ± 0.8	3.1 ± 0.5
	Ra (AUC)	1.17 ± 0.20	1.34 ± 0.22	2.17 ± 0.15
	Ra (C_max_)	1.14 ± 0.31	1.37 ± 0.51	1.85 ± 0.21

^a^ Median (minimum, maximum).

### Safety analysis

The numbers and percentages of subjects who experienced AEs are summarized in Tables 4, 5.

**TABLE 4:** Summary of adverse events at each dose level in the single-dose study.

System organ class preferred term	1 mg (N = 2)	2 mg (N = 6)	5 mg (N = 6)	10 mg (N = 6)	17 mg (N = 6)	25 mg (N = 6)	33 mg (N = 6)	Placebo (N = 12)
Total adverse event (%)	1 (50.0)	2 (33.3)	5 (83.3)	4 (66.7)	5 (83.3)	5 (83.3)	6 (100.0)	8 (66.7)
Metabolism and nutrition disorders (%)								
Hypokalemia	0	0	0	0	0	0	0	1 (8.3)
Investigations (%)								
Fecal occult blood (positive for transferrin)	0	1 (16.7)	2 (33.3)	1 (16.7)	0	1 (16.7)	2 (33.3)	2 (16.7)
Electrocardiogram ST segment abnormal	0	0	2 (33.3)	0	0	0	0	0
Erythrocyte sedimentation rate elevated	1 (50.0)	0	0	0	0	0	0	0
White blood cell count decreased	0	0	1 (16.7)	0	0	0	2 (33.3)	3 (25.0)
Neutrophil count decreased	0	0	1 (16.7)	0	1 (16.7)	2 (33.3)	2 (33.3)	1 (8.3)
The proportion of neutrophils decreased	0	0	0	0	0	4 (66.7)	5 (83.3)	0
Alanine aminotransferase increased	0	0	0	0	0	1 (16.7)	0	0
Triiodothyronine decreased	0	0	0	0	0	0	1 (16.7)	0
Thyroid stimulating hormone elevated	0	0	0	0	0	0	1 (16.7)	0
Fibrinogen decreased	0	0	0	0	0	0	1 (16.7)	1 (8.3)
Glomerular filtration rate decreased	0	0	0	0	0	0	0	1 (8.3)
Creatinine increased	0	0	0	0	3 (50.0)	0	0	1 (8.3)
Positive for urine protein	0	0	0	0	0	0	1 (16.7)	0
Gastrointestinal disorders (%)								
Dental ulcer	0	0	1 (16.7)	0	0	0	0	1 (8.3)
Nausea	0	1 (16.7)	0	0	0	0	0	0
Renal and urinary disorders (%)								
Polyuria	0	0	2 (33.3)	3 (50.0)	4 (66.7)	0	2 (33.3)	3 (25.0)
Blood and lymphatic system disorders (%)								
Anemia	0	0	1 (16.7)	0	0	0	0	0
Infections and infestations (%)								
Bacterial tonsillitis	0	0	0	0	0	0	1 (16.7)	0

**TABLE 5:** Summary of adverse events at each dose level in the multiple-dose study.

System organ class preferred term	5 mg QD (N = 8)	10 mg QD (N = 8)	5 mg BID (N = 8)	Placebo (N = 6)
Total adverse event (%)	6 (75.0)	5 (62.5)	8 (100)	6 (100)
Investigations (%)				
Fecal occult blood (positive for transferrin)	4 (50.0)	1 (12.5)	2 (25.0)	2 (33.3)
The proportion of neutrophils decreased	0	3 (37.5)	1 (12.5)	0
Creatinine increased	2 (25.0)	1 (12.5)	1 (12.5)	0
Positive for urine protein	0	1 (12.5)	0	0
Nervous system disorders (%)	1 (12.5)	0	0	0
Dizziness	1 (12.5)	0	0	0
Gastrointestinal disorders (%)				
Mucositis oral	0	3 (37.5)	0	3 (50.0)
Dry mouth	0	1 (12.5)	0	0
Constipation	0	0	2 (25.0)	1 (16.7)
Renal and urinary disorders (%)				
Polyuria	4 (50.0)	4 (50.0)	8 (100)	4 (66.7)
Infections and infestations (%)				
Pharyngitis	0	1 (12.5)	0	0
Bacterial tonsillitis	1 (12.5)	0	0	0
Respiratory, thoracic and mediastinal disorders (%)				
Epistaxis	0	0	0	1 (16.7)

QD: once daily, BID: twice daily.

In the single-dose study, 58 AEs occurred in 28 (73.7%) of the 38 subjects who received 1–33 mg WXFL10203614. The most common AEs included polyuria (11/28.9%), the proportion of neutrophils decreased (9/23.7%), fecal occult blood (positive for transferrin) (7/18.4%), neutrophil counts decreased (6/15.8%), white blood cell count decreased (3/8.0%) and creatinine increased (3/8.0%), all deemed related to WXFL10203614 before unblinding. 20 AEs were reported in 8 (66.7%) of the 12 subjects who received placebo, mainly including white blood cell count decreased (3/25.0%), polyuria (3/25.0%) and fecal occult blood (positive for transferrin) (2/16.7%). All AEs were mild or moderate and resolved spontaneously.

In the multiple-dose study, 28 AEs occurred in 11 (68.8%) of the 16 subjects in the 5 and 10 mg QD groups. The most frequency AEs were polyuria (8/50.0%), fecal occult blood (positive for transferrin) (5/31.3%), creatinine increased (3/18.8%), mucositis oral (3/18.8%) and the proportion of neutrophils decreased (3/18.8%), respectively. Meanwhile, 21 AEs occurred in all subjects (8/100%) who received 5 mg BID WXFL10203614, mainly including polyuria (8/100%), fecal occult blood (positive for transferrin) (2/25.0%) and constipation (2/25.0%). All AEs were considered to be related to WXFL10203614 before unblinding. However, 15 AEs occurred in the placebo group (6/100%), which was similar to WXFL10203614 groups, such as polyuria (4/66.7%), mucositis oral (3/50.0%), fecal occult blood (positive for transferrin) (2/33.3%). All AEs were mild and subsided without treatment.

There were no AEs, Serious Adverse Events (SAEs) or deaths leading to withdrawal from the single- and multiple-dose study. All AEs were reported to the Ethics Committee of Wuxi people’s hospital.

## Discussion

As far as we know, none of the domestically produced JAK1 inhibitors are marketed in China. It is the first-in-human clinical trial of WXFL10203614, a potent and selective JAK1 inhibitor, conducted in healthy Chinese subjects. The study showed that WXFL10203614 was well-tolerated after a single dose and multiple doses. The most frequent AEs were polyuria, the proportion of neutrophils decreased, fecal occult blood (positive for transferrin), neutrophil count decreased, white blood cell count decreased and creatinine increased, most of which were more common with the higher doses, and have previously been reported in healthy subjects or patients treated with JAK inhibitors (Shi et al., 2014; Taylor et al., 2017; Li et al., 2022). As for polyuria and fecal occult blood (positive for transferrin), they were not reported in JAK inhibitor before and the incidence was not associated with the increasing dose of WXFL10203614. Meanwhile, they were also found in the placebo group. Therefore, the exact reason is unclear yet, which might be related to personal water-drinking habits and diet, and should be explored and paid more attention in future studies. All AEs were mild to moderate without treatment. No SAEs, deaths or clinically significant changes in laboratory tests were observed. Only 1 subject in the 10 mg QD group was discontinued on day 8 for personal reasons.

WXFL10203614 was absorbed rapidly after single dosing with the median T_max_ of 0.48–0.98 h, which was in accordance with the PK profiles of JAK1 inhibitors (Krishnaswami et al., 2015; Peeva et al., 2018; Gao et al., 2022), whereas the elimination rate was slightly slow and t_1/2_ was 9.2–12.4 h. Moreover, MRT_0–t_ was gradually prolonged from 11.6 to 17.1 h with the increase of dose, indicating that there might be a trend of saturable distribution and/or elimination of WXFL10203614. Although PK parameters (C_max,_ AUC_0-t_ and AUC_0–∞_) were elevated in a dose-related manner, the conclusion of an obvious linear correlation could not be made yet. Renal excretion was proved to be the main excretion route of WXFL10203614. The slow elimination of WXFL10203614 might increase AEs caused by drug accumulation when multiple doses were given consecutively. The kidney was the main route of WXFL10203614 excretion, and chronic kidney disease was reported in 10.4% of RA patients before and 89.6% of RA patients after treatment (Fayed et al., 2019). Thus, more attention should be paid to renal function in clinical trials.

In the multiple-dose study, steady-state trough concentrations of WXFL10203614 had achieved after once daily or twice daily dosing for 5 consecutive days. Steady-state PK parameters were also elevated with increasing of the once-daily dose, and AUC _0-t, ss_ and C_max, ss_ in the 10 mg QD group were higher than those in the 5 mg BID group, though the daily dose was the same. In addition, non- and weak accumulations of WXFL10203614 *in vivo* were observed in the 5 and 10 mg QD groups while moderate accumulation in the 5 mg BID group, which were assessed by the calculation method for Ra. The crucial values for non-, weak, moderate, and strong accumulation can be set at Ra < 1.2, 1.2 ≤ Ra < 2, 2 ≤ Ra < 5 and Ra ≥ 5, respectively (Li et al., 2013). RA treatment is a long-term process, the twice-daily regimen may cause drug accumulation of WXFL10203614 *in vivo*, resulting in severe adverse reactions. Thus, the once-daily regimen could be a safer and better way for patients with RA to avoid drug accumulation*.*

As a first-in-human clinical trial, the oral doses of WXFL10203614 were based on the no observed adverse effect levels (NOAELs) originating from the long-term toxicological studies of WXFL10203614 conducted in Sprague Dawley rats and beagle dogs (2 and 6 mg/kg). The NOAELs were finally converted to human equivalent doses (HEDs) according to the guidelines issued by US Food and Drug Administration (US Department of Health and Human ServicesFood and Drug AdministrationCenter for Drug Evaluation and Research, 2005). The safety factor was selected as 20 to decrease the possible risk of WXFL10203614. The maximum starting dose (MRSD) was obtained by dividing the HED by the safety factor. Consequently, 1 mg was calculated as MRSD given to healthy subjects weighing 60 kg. Additionally, in an efficacy study conducted in the rat model of arthritis, the minimal effective dose (MED) of WXFL10203614 was 1 mg/kg converted to HED of 21.5 mg, and the final estimated MRSD was 1.1 mg. To sum up, the starting dose of WXFL10203614 was set at 1 mg, and dose increments were 1, 2, 5, 10, 17, 25 and 33 mg, which was designed according to the modified Fibonacci method and the specifications of WXFL10203614 (1 mg per tablet and 5 mg per tablet) (Simon et al., 1997). The increments of dose for succeeding levels were 100%, 150%, 100%, 70%, 47% and 32%.

Once-daily and twice-daily treatment regimens are two options for treating RA. According to the results of the preclinical pharmacodynamics studies of WXFL10203614, the efficacy of 3 mg/kg WXFL10203614 was equal to 5 mg/kg tofacitinib in the rat model of RA, and the steady plasma concentration of tofacitinib with the therapeutic dose of 5 mg BID. It is speculated that when WXFL10203614 achieved the same clinical efficacy as tofacitinib, the steady-state peak concentration and steady-state exposure should reach 39.23 ng/ml and 287.90 ng*h/mL, respectively. PK model of WXFL10203614 after multiple dosing developed by WinNonlin software which predicted that administration of 5 mg QD, 10 mg QD, 5 mg BID or 10 mg BID WXFL10203614 for 7 consecutive days could achieve this PK profile. Thus, 5 mg QD, 10 mg QD, 5 mg BID and 10 mg BID were chosen as the administration dosages in the multiple-dose study. However, due to the drug accumulation (Ra: 2.17 ± 0.15) of the 5 mg twice-daily regimen, the 10 mg BID study was not performed.

The present study will help to optimize the WXFL10203614 dosing regimen for the phaseⅡstudy of WXFL10203614 in patients with moderate to severe active RA (Registration Number: CTR20202,463), and the drug-drug interaction (DDI) of WXFL10203614 with methotrexate in patients with RA (Registration Number: CTR20212,562) to evaluate the efficacy, safety and DDI of WXFL10203614 better.

## Conclusion

WXFL10203614, the potential selective JAK1 inhibitor, was well-tolerated and safe in healthy Chinese subjects, and plasma exposure increased in a dose-related manner after single-dose and once-daily administration, supporting further evaluation of WXFL10203614 in patients with RA.

## Data Availability

The original contributions presented in the study are included in the article/Supplementary Material, further inquiries can be directed to the corresponding author.
